# Mamma Mia: A Feasibility Study of a Web-Based Intervention to Reduce the Risk of Postpartum Depression and Enhance Subjective Well-Being

**DOI:** 10.2196/resprot.2659

**Published:** 2013-08-12

**Authors:** Silje Marie Haga, Filip Drozd, Håvar Brendryen, Kari Slinning

**Affiliations:** ^1^National Institute of Infant Mental HealthCenter for Child and Adolescent Mental Health, Eastern and Southern Norway (RBUP)OsloNorway; ^2^The Norwegian Centre for Addiction Research, Institute of Clinical MedicineFaculty of MedicineUniversity of OsloOsloNorway

**Keywords:** pilot project, Internet, early intervention, depression postpartum, health promotion, well-being, eHealth

## Abstract

**Background:**

Currently, 10-15% of women giving birth suffer from symptoms of postpartum depression. Due to a lack of knowledge of this condition and the stigma associated with it, as well as few treatment options, a large proportion of postpartum women with depression remain untreated. Internet-based interventions have been found effective in treating depression, anxiety, phobias, and addictions. Hence, we developed such program (“Mamma Mia”) with the aim of reducing the risk for postpartum depression and enhance subjective well-being. Mamma Mia is based on positive psychology, metacognitive therapy, and couples therapy. It starts in gestational week 22, and lasts until 6 months after birth. During pregnancy, Mamma Mia is delivered weekly (every Monday). After birth, Mamma Mia is delivered three times per week for six weeks. The remaining weeks, the program is delivered more sporadically. In total, Mamma Mia consists of 44 sessions. The program is individualized, interactive, and tunneled (ie, the user is guided through the program in a pre-determined manner).

**Objective:**

The purpose of the present study was to pilot test the intervention in order to assess the feasibility and acceptance among program users.

**Methods:**

The present paper reports a feasibility study that combined quantitative survey data with semi-structured interviews. Participants (N=103) were recruited via hospitals, well-baby clinics, and Facebook. Due to time constraint in completing the current study, our results were based on participation in one of the two phases: pregnancy or maternity. Participants in the pregnancy phase were surveyed 4 and 8 weeks after intervention enrollment, and participants in the postnatal phase were surveyed 2 and 4 weeks after intervention enrollment. The survey assessed perceived usefulness, ease-of-use, credibility, and unobtrusiveness. All measures were filled in by participants at both measurement occasions. Data were analyzed by running descriptives and frequencies with corresponding percentages. Binomial tests were carried out to investigate whether demographics differed significantly from a 50/50 distribution. Paired sample t tests were used to examine differences between time 1 and 2. Four participants were interviewed in the qualitative follow-up study, where they were given the opportunity to address and elaborate on similar aspects as assessed in the survey.

**Results:**

More than two-thirds of users found Mamma Mia to be of high quality and would recommend Mamma Mia to others. By far, most also found the amount of information and frequency of the intervention schedule to be appropriate. Mamma Mia was perceived as a user-friendly and credible intervention.

**Conclusions:**

Overall, the user acceptance of Mamma Mia was good and our findings add to the feasibility of the program. The effect of Mamma Mia on depression and subjective well-being will be evaluated in a large randomized controlled trial, and if found to be effective, Mamma Mia could serve as a low-threshold prevention program.

## Introduction

### Postpartum Depression: Prevalence, Risk Factors, and Treatment

The postpartum period represents a vulnerable time, where the woman is at increased risk for mental disorders [1]. Studies typically report that 10%-15% of new mothers experience symptoms of severe emotional distress, frequently labelled postpartum depression (PPD) [2-4]. Postpartum depressive disorders vary in severity; ranging from the mildest kind seen in postpartum blues to moderate or major depression, to the most severe cases known as postpartum psychosis [3]. While only 0.2% of new mothers experience postpartum psychosis, postpartum blues affect as many as 50%-80% of postpartum women and it is thus considered to be a fairly “normal” phenomenon. Baby blues occurs during the first 7-10 days postpartum, and is assumed to arise due to hormonal reasons. Eventually, it wanes without treatment, especially with the support of family and friends and with the reassurance of health personnel that this reaction is quite normal [2]. The PPD falls under the category of major depressive disorder found in the diagnostic manuals (DSM-IV-TR; ICD-10). According to the manuals [4,5], description, symptoms, course, and outcomes of PPD are similar to major depressive disorder. The only difference is the time of its occurrence. The depression strikes in a woman’s life when she is expected to be as happy as can be, which obviously makes the experience of the depression particularly arduous. In order to fulfill a diagnosis of PPD, one must experience a period of at least 2 weeks of depressed mood or loss of interest in almost all activities, as well as experiencing at least four of the following symptoms: change in appetite and weight, sleep, and psychomotor activity; decreased energy; feelings of worthlessness or guilt; difficulty thinking, concentrating, or making decisions; and recurrent thoughts of death or suicidal ideation, plans, or attempts [4].

Factors that put women at elevated risk for PPD include a personal history of depression, family history of depression, negative life events, partner conflicts or low relationship satisfaction, low levels of social support, and certain baby characteristics [6,7]. The most common symptoms of PPD are tearfulness, feelings of hopelessness, inadequacy, guilt, inability to cope with and feel joy over the arrival of new baby, agitation and anxiety, loss of appetite, poor concentration and memory, sleep disturbances, fatigue, social isolation, and suicidal ideation [8]. Women who suffer from PPD are less capable of carrying out maternal duties, such as engaging in important developmental activities with the baby, like playing and talking, which may influence the child’s cognitive, and socioemotional development [9], as well as the infant’s attachment style [10]. Men have an increased risk of depression when their partner is depressed [11], and children of depressed fathers are at increased risk of behavior problems [12]. Finally, many women risk experiencing less severe on-going symptoms long after the depression is considered over, and they are at risk for recurring depression [13]. The personal cost as well as the cost to society caused by PPD is large; thus, illustrating the pressing need for effective prevention and early intervention.

According to the National Institute for Health and Clinical Excellence (NICE) guidelines [14], effective treatment of depression is to provide immediate support and information, problem-solving, and self-help. Several studies have also found cognitive-behavioral therapy [15], cognitive treatments [16], and metacognitive therapy [17] to be effective. However, a large proportion of those who suffer from PPD do not receive treatment, in part due to the stigma associated with the disorder as well as limited access to effective treatments [18]. Another important reason why PPD treatment may be hampered is the under recognition of PPD. The under recognition is not only due to the brief consultations with the medical health professionals, but is also due to the difficulty in distinguishing PPD symptoms from normal symptoms seen in new mothers, such as tiredness, change in eating habits, difficulty with concentration and sleeping. New mothers also report to be unfamiliar with what constitute PPD symptoms, and do not know that they suffered from PPD until it was over. All these factors combined cause many people to suffer “in silence’’. In sum, barriers to effective treatment including lack of resources and trained providers and social stigma associated with PPD [19] suggest a need for innovative approaches to public health prevention and promotion.

Many Web-based interventions have been designed and documented to be effective in treating various mental problems including depression [20], anxiety [21], and stress [22]. Studies document that people with these mental disorders greatly benefit from Web-based interventions [23,24] and that Web-based interventions represent a low threshold service with a high reach [25]. More recently, two studies found support for the feasibility and usability of Web-based interventions targeting PPD [26,27], but there exist no randomized trials testing the efficacy of PPD interventions. Web-based interventions have been found successful in reaching women of lower socio-economic status, a group typically found to be harder to reach [28]. It is also noteworthy that young women (ie, women in childbearing age) are the ones who use Internet most frequently when it comes to acquiring health-related information [29]. A Web-based intervention can thus both prevent the development of depressive symptoms as well as reach out to the many people that suffer “in silence’’, and it seems promising considering that many people prefer first line services (ie, local- and home-based services) rather than regional or specialist health care services.

### The Web-Based Intervention: Mamma Mia

#### Overview

Mamma Mia is an automated/unguided Web-based self-help intervention. The aim of Mamma Mia is threefold: (1) to prevent PPD, (2) to treat pregnant women/new mothers with mild-to-moderate symptoms of depression, and (3) enhance subjective well-being. Overall, Mamma Mia consists of 3 phases (see Table 1). The pregnancy phase starts in gestational week 22 and ends in week 40 (estimated due date). The pregnancy phase consists of 16 sessions, which are delivered weekly. The maternity phase starts when the infant is 1-2 weeks old and lasts for 6 weeks. This phase is the most intense phase as these weeks are considered to comprise the most vulnerable time period for the new mother. Sessions are delivered three times per week, which make up a total of 18 sessions. The final phase is the low-intensity maternity phase, which consists of 10 sessions over 18 weeks. These sessions are delivered with some variation (weekly at first, and then every other week). In total, Mamma Mia consists of 44 sessions over a period of 11 months. For each session, the user receives an email with a hyperlink. By activating the link, the user gains access to Mamma Mia and proceeds in a predetermined/tunneled sequence of Web pages (ie, the tunnel information architecture) [30]. In terms of delivery methods and information architecture, Mamma Mia is similar to previous eHealth programs, and thus represents a well-tested approach to treatment [31-34].

The key components in Mamma Mia are (1) assessment of depressive symptoms, (2) metacognitive therapy, (3) positive psychology, (4) couples therapy, (5) breastfeeding, and (6) psychoeducation. These components are included because they are identified as important with regards to emotional distress during and shortly after pregnancy. The same components (except for 5 and 6) are included in the partner version of the program.

#### Assessing Depressive Symptoms

The most common screening-tool for PPD is the Edinburgh Postnatal Depression Scale (EPDS), which assesses depressive symptoms during the last 7 days [35]. The program will monitor the participants’ level of depressive symptoms over time (as measured by the EPDS). Depressive symptoms will be assessed on three occasions during pregnancy and four times after birth. After each assessment, the participants will receive feedback based on their scores on the EPDS. The feedback is divided into three categories: (1) when there are no depressive symptoms, (2) when there are some depressive symptoms, and (3) when the score is indicative of severe depression. If the participant’s score suggest the presence of some/many depressive symptoms, the program will recommend the user to speak to someone they trust about how they are feeling, and if necessary, seek professional help (the program suggests where one can seek professional help). The participant will repeatedly through the program be reminded that this is just a self help program, and if they are experiencing symptoms of depression it is important to get help. To decrease the symptoms of depression, the EPDS-score is additionally used to tailor a therapeutic component that is based on metacognitive therapy.

#### Metacognitive Therapy

Metacognitive therapy [17] is a recent development in understanding and treating mental health problems, which has been proven effective in treating depression [36]. The approach is based on a specific theory proposed by Wells and Matthews [37]. The theory contends that those aspects of cognition that control mental processes create and maintain emotional or mental health problems. This mechanism leads to patterns of worry, rumination, threat monitoring, high conceptual activity, and counterproductive coping behaviors, and it is referred to as the cognitive attentional syndrome. In contrast to cognitive therapy, metacognitive therapy does not deal with the content of specific negative thoughts, feelings, or beliefs (eg, “I am worthless and incompetent”). Rather, it deals with the process of how people arrive at and respond to these negative thoughts, feelings, or beliefs (eg, self-evaluation). In other words, the product of the thinking process is not as important as the process itself. It is an inflexible and recurrent thinking style in response to negative thoughts, feelings, or beliefs that causes problems and needs to be treated. In consequence, a key aspect in metacognitive therapy is therefore to help people experience detached mindfulness, which is a disengaged and objective form of awareness in response to thoughts, feelings, and beliefs. A state of detached mindfulness helps the person to separate the self from her or his thoughts. Thus, the present program educates users about mindfulness exercises to reduce the functions of thoughts [38]. One such exercise is turning negative thoughts track, which is based on the acceptance and commitment therapy [39], and uses a combination of acceptance and mindfulness to help people distinguish themselves from thoughts, feelings, sensations, and memories. Other concrete tasks that aim to enhance mindfulness include “the body scanning” exercise and “the gong” exercise. Both tasks are based on audiotapes that instruct the person to try to visualize and focus on either a sound (the gong) or a marble travelling up and down on one’s spine. Thoughts can come and go, one is only to register that they are there, not evaluate them.

**Table 1:** Overview of the online-sessions of Mamma Mia across program phase.

	Pregnancy phase	Maternity phase (high intensity)	Maternity phase (low intensity)
Time period	Gestational week 22-40	2-8 weeks postpartum	9-26 weeks postpartum
Frequency	1 session per week	3 sessions per week	Variation^a^
Number of sessions^b^	16	18	10

^a^From 9-14 weeks postpartum, there is 1 session per week. From week 14-18, there is 1 session every other week. Then there is one session 22 weeks postpartum, and the final session is 26 weeks postpartum.

^b^Total number of sessions is 44.

#### Positive Psychology

Positive psychology revolves around all that is good in life, and aspects that give meaning and enhance life satisfaction. Concrete tasks aimed at enhancing positive emotions, engagement, and a sense of meaning/purpose has been developed [40]. The basic idea for the different tasks is the assumption that the effect of positive emotions extends beyond the immediate moment. Peterson and Seligman [41] argue that engaging in the tasks may enhance well-being, a sense of skill, efficiency, mastery, mental hygiene, as well as a person’s social network. That is, a person’s fundamental needs may be strengthened, which in turn may lower the risk of mental disorders. Indeed, several studies have demonstrated that various “happiness-tasks” have not only enhanced a person’s sense of life satisfaction, but also reduced the prevalence of depression [42,43]. Seligman et al [44] argue that these studies suggest efforts aimed at preventing/treating depression would benefit from including components based on positive psychology. One of the crucial ingredients in the present program is exactly that. Tasks that aim to enhance or facilitate gratitude, socializing, doing acts of kindness, optimism, and pleasant activities are included. The different tasks are described in some detail below.

The gratitude component involves assignments such as counting one’s blessings, writing down 3 good things that happened during the day, writing a letter of gratitude, and saying “thank you” more often than usual. In order to increase socialization and thus strengthen the social network, which in turn facilitates social support, one is encouraged to initiate social interaction with friends and relatives. Assignments include calling a friend and inviting him/her out and mapping one’s social network. One is also advised on how to get new friends, and strengthening existing relationships (eg, find out about a friend’s plan and follow-up the next week by asking how it went, or give compliments to a partner or friend). In terms of acts of kindness, one is encouraged to do acts of kindness to others, keeping track of such acts, and plan them ahead of time. Examples of kind acts are provided, while one is also encouraged to figure out such acts for oneself. The “best possible self” exercise is intended to increase optimism [45,46]. Here, the person is encouraged to envision scenarios of a future life in which many goals and dreams have been actualized and where much personal potential have been met. Assignments in the coming week will ask the person to elaborate on the scenarios, to recognize what one has already achieved that is in line with the best possible life scenario, challenge and turn around obstructive thoughts, and break major goals down into achievable sub-goals and milestones. The final positive psychology task is based on cognitive behavioral therapy [47], and is intended to facilitate the engagement in pleasant activities. The program starts by prompting the person to compile a list of activities that make her/him feel good. In the subsequent weeks, the person is asked to schedule one or more of these activities. She or he is also asked to determine a goal for one of the activities. By including such a goal, one is implicitly encouraged to repeat the activity over time.

#### Couple’s Therapy

A recent study suggests relationship dissatisfaction to be the strongest predictor of maternal emotional distress during pregnancy [48,49]. Hence, a unique component of the program is dedicated to enhancing communication between partners and facilitating healthy conflict resolution, which in turn is expected to improve relationship satisfaction. The couple’s therapy is mainly based on a course that has been evaluated as an online program [50]. The course is based on cognitive behavior therapy. Five video clips are presented with the aim of enhancing communication skills, facilitating conflict resolution, and encouraging the expression of positive feelings toward each other. Following each video clip, one is encouraged to make use of the techniques that have been introduced. An example of a task is to let one’s partner know when he or she made you feel appreciated; this task is intended to facilitate the expression of positive emotions between partners. Another task that targets healthy conflict resolution is “the active listening” task. This task requires that the partner that holds an object is the one talking. The other partner listens. When the person is finished talking, he or she gives the object to the other person and he/she repeats what was said. This task is intended to encourage listening and to raise awareness in terms of how one typically and ideally communicates in a relationship.

#### Breastfeeding

Research suggests a relation between breastfeeding and depressive symptoms [51-55]. Formative research done prior to the development of Mamma Mia aimed to discover the topics that were most important with regards to well-being and depressive symptoms [56,57]. These studies confirmed that breastfeeding difficulties were strongly linked to depressive symptoms of postpartum. Hence, a component in the program focuses on breastfeeding, both normalizing difficulties associated with it, providing useful advice and assistance, as well as informing of the optional use of substitute.

#### Psychoeducation

Every session in the program includes information regarding topics such as emotional lability in pregnancy, changes and challenges in the partner relationship, the importance of social support, the fetus and the infant’s development, birth etc. Short video clips are available to demonstrate and explain the infant’s state regulation system (sleep-wake cycles), and this information is integrated with issues related to the infant’s capacity to sleep and rest, soothability, interaction/communication etc. The intention is that the program will provide the woman with relevant information adjusted to her own progress through pregnancy and after the child is born.

### The Present Study

Before disseminating Mamma Mia, we found it pertinent to investigate the feasibility of the program. Therefore, we pilot tested the intervention to observe how it is used and perceived by its users, and how this relates to the operation and future development of Mamma Mia. Consequently, the objectives with the present study were to (1) examine user acceptance of Mamma Mia, (2) examine how it was perceived among end-users, and (3) identify potential issues with use, acceptance, and program-specific needs that might provide added value to Mamma Mia and its operation and future development.

## Methods

### Survey Design and Data Collection

#### Overview

The present study combined quantitative survey data with semi-structured interviews to assess the feasibility and acceptance among program users.

Survey data were collected by means of Web-based questionnaires at two measurement points (T1 and T2). Due to time constraint in completing the current study, our results were based on participation in one of the two phases: pregnancy or maternity. Participants in the pregnancy phase were surveyed 4 and 8 weeks after intervention enrollment, and participants in the postnatal phase were surveyed 2 and 4 weeks after intervention enrollment. Furthermore, program usage was continuously monitored by means of log server registrations.

#### Measures

##### Perceived Usefulness

Six items were rated on a scale from 1-7 in order to assess a person’s belief that using Mamma Mia is useful and helps increasing well-being [58].

##### Perceived Ease-of-Use

Four items were rated on a scale from 1-7 to measure a person’s belief that using Mamma Mia is free of effort [58].

##### Perceived Credibility

Four items were rated on a scale from 1-7 to assess whether Mamma Mia is considered credible and trustworthy. User Satisfaction is measured in terms of how users rate the overall quality and satisfaction of the program, and whether they would recommend it to others. Four items were rated on a 1-7 scale [59].

##### Unobtrusiveness

Four items were rated on a 1-7 scale to assess whether users have the opportunity to use Mamma Mia seamlessly as part of their daily routines. Estimated means of all the measures were reported in Table 4 [60].

Technical problems were measured using a single yes/no option (“Have you experienced any technological problems in using Mamma Mia?”). Participants that responded “yes”, were provided an additional open-ended question, “Please describe the technological problems you have been experiencing?”

Improvements were assessed using a single open-ended question, “If you could improve anything in Mamma Mia, what would that be?”

### Recruitment and Participants

Participants were recruited at 5 health care clinics and 2 hospitals. Here, midwives/public health nurses handed out a brochure and offered to try Mamma Mia. We recruited primarily women, and their partners through the women. The reason why we did it this way is because women often go to the follow-ups at the well-baby clinics and hospitals by themselves. Potential participants had to take home the brochure and send an email to the administrator of the program to register for participation. Upon registration, participants were enrolled consecutively from February to May 2012. Participants were also recruited on Facebook (30% of the total sample). In order to be included in the study, one had to be in gestational week 22 or 2-3 weeks after birth, or one had to be the partner of a women in gestational week 22 or who had given birth within the last two weeks. The two inclusion criteria we applied were that all participants had to be 18 years or older, and they had to be able to read and understand Norwegian.

A total of 103 users were recruited from February to May in 2012. By far, most participants were female (82%) and the mean age was 31.4 years (SD 4.3; range 20-41 years). Female participants were on average slightly younger (mean 31.1, SD 4.4) than male participants (mean 33.4, SD 4.0). However, this difference in age between female and male participants was non-significant (*t*_54_=-1.1, *P*=.28). Furthermore, binomial tests of 50/50 proportions (all *P* values, *P*<.02) confirm that most participants were ethnically Norwegian, had 4-5 years of college or university education, currently employed, and had no previous children (*P≤*.02). Most participants who were recruited were in their pregnancy and multinomial tests of equal categories show further that most participants were currently in a relationship (*P≤*.01). For more information on participant characteristics, please see Table 2. Missing data ranged from 4% (41/103)-32% (33/103).

### Interview Design and Data Collection

A total of 103 participants were recruited to test the usage of Mamma Mia, among which a random subset of 10 was invited to take part in an interview. Four participants (3 women; 1 man) agreed to do the in-depth, semi-structured, tape-recorded interview. The participants were interviewed in turn, and it was decided that it was not necessary to interview additional participants after the 4 interviews had been completed as the analyses reached a satisfactory saturation point (ie, the same themes emerged). An interview schedule was developed with open-ended questions with prompts and follow-up questions employed to elicit a breadth and depth in responses [61]. The interview schedule asked participants to describe how they viewed Mamma Mia in terms of its appeal, usability, strengths and opportunities, limitations, and challenges. The interview schedule also addressed the participants’ fidelity to the program, and why there had been a high/low degree of fidelity. Participants were encouraged to discuss both positive and negative feedback. Participants were also encouraged to think aloud in terms of how to best market the program, and the final question was an open question, where participants could raise issues that had not been addressed in the interview. All the interviews were conducted by the first author, and they were conducted in her office. They lasted approximately 1 hour. The data were transcribed verbatim.

### Participants

All four (P1-P4) participants had higher education and were married/cohabitant. Three participants had previous children. Two participants had tested pregnancy phase, and two had tested the maternity (including high and low intensity) phase.

**Table 2:** Participant characteristics (N=103).

Variable		Frequency	Percentage
**Gender**			
	Female	85	82.5
	Male	14	13.6
**Marital status**			
	Single	6	5.8
	Co-habitant	31	30.1
	Married	34	33.0
**Education **			
	≤ 1-3 years college or university degree	22	21.4
	≥ 4-5 years college or university degree	48	46.6
**Occupational status**			
	Employed	61	59.2
	Unemployed or student	10	9.7
**Children**			
	No children	46	44.7
	1 or more children	25	24.3
**What part of Mamma Mia was accessed **			
	Pregnancy	75	72.8
	Maternity	23	22.3
**Parental role by program version **			
	Mother, pregnancy	64	62.1
	Mother, maternity	20	19.4
	Father, pregnancy	10	9.7
	Father, paternity	4	3.9

## Results

### The Survey

#### Program Usage

In total, 78.6% (81/103) participants were engaged with Mamma Mia, while 21.4% (22/103) participants did not initiate the use of Mamma Mia. In the pregnancy phase, the average number of completed program days was 7.4 (SD 6.9). Median number of completed program days was 6. In the maternity phase, the average number of completed program days was 11.5 (SD 11.8). The median number of completed program days was 5. The program lasted for 11 months. Due to time constraint in completing the current study, however, we chose to study adherence within a limited time frame. Consequently, users have not been given the chance to complete the program. Thus, usage data may not accurately reflect adherence.

#### Survey Response Rates

The response rates varied from 25% (6/24)-58.6% (44/75) (see Table 3 for details). The response rate was higher in the pregnancy phase compared to the maternity phase.

#### User Acceptance

Overall, 65% (67/103) of responders rated Mamma Mia to be of high quality. Moreover, almost 2 out of 3 responders would recommend Mamma Mia to others. A total of 43% (44/103) reported that the information presented in Mamma Mia was relevant to them. In terms of the quantity of information in each session (ie, program day), users reported that the amount of information was appropriate. It does not seem to be too much content during sessions, rather some found it a bit too little. In the pregnancy phase of Mamma Mia, 78% (58/75) reported that one session per week was appropriate, while the rest found one session per week to be too rare. In the maternity phase, 67% (16/24) found three sessions per week to be appropriate, while 33% (8/24) found it a bit too much.

Table 4 illustrates how participants rated Mamma Mia in terms of usefulness, ease-of-use, credibility, unobtrusiveness, and satisfaction. The theoretical range goes from 1 through 7 for all five scales. Generally, ratings were high, indicating good user acceptance. Paired sample *t* tests were performed to compare ratings across time (T1 and T2), and results suggested that the ratings were stable over time. Therefore, Table 4 reports the figures from T1 only.

#### Improvements

**Figure 1:**
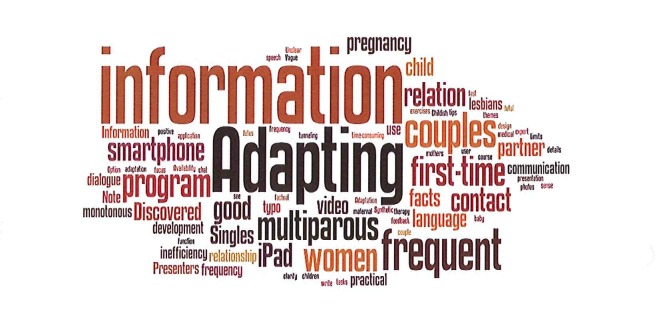
De-contextualized word cloud analysis of suggested improvements.

A total of 47 improvements were suggested by the participants. Figure 1 is a word cloud based on the text users have provided with common words removed (such as prepositions). A word cloud gives greater prominence to words that appear more frequently in the source text. As can be seen, words like “information”, “adjustment”, “platform”, “independence”, and “multiparous” were the most prominent. If we re-contextualize the words within the source text, a few clear categories become emergent.

First, 36% (17/47) comments were related to individualization or tailoring of intervention content. Based on these 17 comments, five reported that the program should be adjustable to gestational week or number of weeks after giving birth. Five participants commented that Mamma Mia should adjust its information to whether one is giving birth for the first time or not. Finally, 6 participants also noted that Mamma Mia should adjust the couples’ therapy so that (1) users without partners can skip the couple’s therapy and (2) that the couples’ therapy can be repeated so partners more easily can manage to follow it together. Regarding (1), one participant actually reported feeling “down” when faced with the couples’ therapy as it merely acted as a reminder about a recent sensitive event (ie, most likely a breakup). Although such cases may not occur very frequently, they pose a real threat to the well-being of the individual and act counterproductive to the objective of Mamma Mia. Regarding (2), it is not exactly clear, based on participants’ comments, what actions can be taken to help couples follow the couples’ therapy together. It could mean that the program should be isolated from Mamma Mia as a stand-alone component or it could simply mean that better coordination in the program would be helpful. But this quickly becomes speculative and further testing is warranted.

Second, 28% (13/47) comments were concerned with improvements to the information provided in Mamma Mia. It seems that users have a two-fold opinion. On the one hand, some users request more information, especially about the baby in utero and about mothers’ emotional and physical development during pregnancy. On the other hand, some information seems redundant or familiar, especially for women who already have children. The latter may be an important cue for increasing adherence. In a recent study by Tonkin-Crine and colleagues [62], they interviewed participants to identify reasons for not engaging with a Web-based intervention for chronic illness. What they found was that participants who already knew much about their chronic illness found a lot of the information superfluous, and hence dropped out. Participants suggested that more information should be optional or adjusted according to the level of knowledge of the participant, which inevitably leads to some form of individualization or tailoring.

The third emergent category is related to technological development. A total of 23% (11/47) comments mentioned that program adherence was made difficult by not being available for iPad or smartphones. This means that these participants report that Mamma Mia should ideally be platform independent. Platform independence ensures the accessibility and ubiquity of Mamma Mia as participants can take the program on-the-go or wherever they are. In conclusion, we have identified three main categories of suggested improvements which account for 87% of the variation in participants’ responses. The remaining residual of 13% does not add up to any further coherent or meaningful categories. Typically, the residual consists of individual responses such as “discovered typos” or “option for chatting with health personnel”.

**Table 3:** The response rate across program phase and assessments.

	Pregnancy phase (n=75)	Maternity phase (n=24)
T1	44 (58.6%)	8 (33.3%)
T2	31 (41.3%)	6 (25%)

**Table 4:** User acceptance of Mamma Mia.

Variable	Mean	SD
Perceived usefulness	4.2	1.3
Perceived ease-of-use	6.1	1.0
Perceived credibility	5.8	0.9
Unobtrusiveness	5.1	1.4
User satisfaction	4.6	1.5

### Reflective Practice and Participant Feedback

The first lesson from the present study is that not all participants seem to read information about Mamma Mia very carefully during recruitment. It is estimated that roughly 10% of the participants that were recruited, had passed the gestational week 22 or were more than 2-3 weeks passed giving birth. Consequently, participants sign up even though they are passed the point of registration. This results in “confusion”, “misunderstandings”, and questions about “where can I adjust the program to my gestational week?” At least two participants also had to be re-registered from the prenatal phase to the postnatal phase of Mamma Mia, because they signed up late in their final trimester.

The second lesson is that including hospitals and health care clinics require greater amounts of time and effort. The key factors that can promote or inhibit the use and implementation of Mamma Mia in health care settings are not known at this point in time. However, the overall impression during recruitment was that hospitals and health care clinics experienced difficulties recruiting the numbers expected and needed. Only 61.2% (63/103) participants were recruited at hospitals and health care clinics despite the fact that 925 brochures were handed out and 9 meetings were held about Mamma Mia, at the very same hospitals and health care clinics. Consequently, RBUP (Centre for Child and Adolescent Mental Health) started recruiting participants online after the recruitment had started at hospitals and health care clinics, and with less time and less effort they managed to recruit 35% (36/103) participants. Also, due to the slow recruitment, the question “Where were you recruited for the user survey of Mamma Mia?” was included late into the recruitment period. Out of 7 responses, only 1 reported being recruited at a health care clinic. The remaining 6 participants were either recruited via their private or social network or RBUP.

### Data Analysis and Results

#### The Interviews

##### Overview

We were interested in the women’s qualitative experience of Mamma Mia, and in the issues they themselves raised in relation to it. The analyses relied upon organizing sections of the data into recurrent themes [61]. In accordance with Braun & Clarke [63], our approach is based on a thematic analysis, as opposed to a theoretical analysis; our themes were identified in an inductive manner, making the themes strongly linked to the data; and we took a semantic approach in that we did not look for a deeper meaning beyond what the participant said. Furthermore, we followed Kissling’s recommendation [64] to let the data itself suggest names for the themes, and took direct quotes from the transcripts to illustrate the kind of data classified by each theme. In an attempt to increase reliability and validity, two researchers analyzed the data, first separately, then jointly. Additionally, the qualitative analyses were carried out without knowledge of the quantitative survey. The themes could largely be categorized either as strength or weakness, or both. Thus, the findings are presented below in terms of strengths and weaknesses for each theme.

##### Strengths

###### Accessibility

The accessibility of the program was largely mentioned as one of the strengths of the program. The opportunity to complete the sessions at home or at work, depending on when one had the time was greatly appreciated. This is in line with previous research [65].

###### Information: Amount and Frequency

Participants appreciated that the information was concise and brief, and it was easy to understand. Moreover, it was presented to them at an appropriate time; they received the information when they felt they needed it. The women enjoyed that the program was organized according to a tunneled sequence; that is, they were guided through the program and had to complete one module before proceeding to the next. As one participant said “to me, the program felt like a safe guidance that contained the information I needed when I needed it” (P1). The frequency of the sessions was found to be appropriate, although it was suggested that the preparation phase of the program could ideally have had more frequent sessions.

###### Psychoeducation

The interviewees expressed enthusiasm with regards to the information and exercises that targeted the relationship with partner and sensitivity with regards to the infant’s signals. As one participant said: “With Mamma Mia, you get the knowledge you won’t get anywhere else” (P2). Moreover, they enjoyed how the program was not a mere channel for information that provides general advice, but it “raised” questions that stimulated to self-reflection and it provided helpful exercises in an interactive manner. These aspects made learning fun, and stimulated to change.

###### Confidence and Credibility

Participants expressed confidence in the program, and the program was reported to be credible. Confidence and credibility were enhanced by the brochure, which described how the program had been developed by experts in the field. It was emphasized in two of the interviews how it was of great importance that health personnel whom they trusted were the ones who conveyed information regarding the program. One participant (P1) expressed how she felt “that the program was developed and provided as part of the public health care system”, and thus she felt that the program was a good thing for her.

###### Fidelity

The major reason for high fidelity was the participants’ curiosity in terms of what was to come in the program. Another reason why participants completed the program was that completing the sessions proved to be break and a “time-out” for multiparous women in a hectic everyday life. As one woman said “the program provided an opportunity to take the time to connect with the baby in my tummy” (P3). Ordinarily they would not have taken the time to focus inward on the fetus, and so the program facilitated contact and attachment with the unborn baby. A final reason for completing the program was the desire to be confirmed; confirmed that she was “doing the right thing” for her baby (P1).

##### Weaknesses

###### Inflexibility

A major limitation had to do with a lack of flexibility. As one participant said, “my main complaint is the inflexibility in terms of when you can start with the program, you should be able to start up regardless of where you are in your pregnancy” (P2). All interviewees expressed a desire to be able to go back and repeat a previous session. The inability to use the program on tablets and smart phones was also mentioned as a major limitation. Finally, because of its emphasis on the relationship with the partner, the program was viewed as targeting the nuclear family, and other family constellations (such as single mothers) would likely not find the program to be adjusted to their needs. As one participant said, “a friend of mine, who is about to become a single mom…she sort of chose to have this baby on her own. She went to Denmark and had a donor. I thought many of the themes in the program would be good for her, but then I thought, no, no, it wouldn’t work, cause the program is really custom-made for the “standard person” for the nuclear family” (P1).

###### Information

Although the information provided throughout the program was largely emphasized as one of the strengths in the program, it was mentioned that the follow-up phase (10 weeks postpartum and onward) could have contained more information regarding the baby’s development and attachment etc.

##### Recommendations for Improvement

###### Accessibility and Fidelity

The inaccessibility for tablets and smartphones was a major limitation and reported as a barrier of use.

###### Expectations

The interviewees were encouraged to describe how the program could be further improved, and expectations emerged as a common topic. Specifically, they wanted the program to focus more on expectations with regards to breastfeeding. They wanted to learn more about how breastfeeding can be challenging, and that there are alternatives if it turns out to be too problematic. Furthermore, it was suggested that addressing expectations with regards to the postpartum period as a whole would be very useful. Many new mothers plan to do too many things after having a baby, and Mamma Mia could therefore serve as a useful reminder that having a baby is both unpredictable and inherently hectic in and of itself.

## Discussion

### Principal Findings

The purpose of the present study was to assess the feasibility and acceptance among program users with regards to the Web-based intervention Mamma Mia, as well as to identify potential issues that could be improved. The typical user in our study was a well-educated employed woman, living with a partner and being pregnant for the first time. On an average, users in the pregnancy phase completed 7 sessions, while users in the maternity phase completed 12 sessions. The user-survey and interviews identified critical internal and external success factors for initiation and continued use of Mamma Mia (ie, factors that promote or inhibit intervention adoption) [66]. Internal factors refer to the strengths and weaknesses internal to the intervention such as its information quality, while external factors refer to the opportunities and threats presented by the external environment to the intervention such as technological changes or lack thereof. Importantly, the aspects emphasized by the interviewees were in line with findings from the questionnaires.

### Program Strengths and Weaknesses

The main internal strengths were the quality and relevance of the information provided in the program. More than two-thirds of users found Mamma Mia to be of high quality and would recommend Mamma Mia to others. By far, most also found the amount of information and frequency of the intervention schedule to be appropriate. Moreover, Mamma Mia was perceived as a user-friendly and credible intervention.

In terms of internal weaknesses, it was mentioned that the program sessions could be more frequent during the pregnancy phase, and in the maternity phase there could be more information regarding the baby’s development and attachment.

### Opportunities and Threats for Real Implementation

Mamma Mia is operated on a stable platform and there were few reports of technical problems. In line with previous research [63], the accessibility of the program in terms of allowing users to complete the sessions at home or at work emerged as an important advantage of the program. Health care workers that conveyed the information regarding Mamma Mia emerged as both an external threat and potential strength. They constituted strength in terms of enhancing the credibility of the program, which in turn made the participants more eager to try the program. However, as described earlier, many health care workers failed to inform potential participants about the program, which posed a fundamental barrier to initiation of the program. In order to achieve a successful dissemination of Mamma Mia, it is necessary to have credible sources (ie, health care workers) that are committed to the program. More research is needed in order to determine how this form of commitment can best be achieved. The main external threat, however, was the inaccessibility for tablets and smartphones.

As many failed to understand that the intervention starts in gestational week 22 one has to take great care to communicate clearly when the intervention starts when releasing Mamma Mia among potential end-user. Communication and marketing plans have to ensure that users are registered at the right time according to gestational week, and one has to carefully plan how to implement Mamma Mia during point of care in health care settings.

### Improvements

Findings suggest a need for improvements in mainly three domains: (1) making Mamma Mia available for iPads and smartphones to increase accessibility, (2) provide more information, and (3) individualization to gestational week, couples versus singles, first-time parents versus second-time parents.

The most common barrier of use was the inaccessibility for *tablets and smartphones.* In turn, improvements were made such that the final version of Mamma Mia is available for iPads and smartphones.

More frequent program sessions during the pregnancy phase as well as more *information* regarding breastfeeding, sleep, child development, and attachment were requested, and all of these requests were taken into account and incorporated in the final revision of the program. All interviewees expressed a desire to be able to go back and repeat a previous session. Thus, the final version of Mamma Mia includes a “personal” home page, which gives an overview of all the sessions one has completed, and all the sessions that are to come. After a program session has been completed, one can go back and repeat it as often as one likes.

In terms of *individualization*, there is a definite long-term goal to adjust Mamma Mia to the needs of single parents, parents who have premature infants, parents with previous children, as well as non-Norwegian speakers. Participants in the present study also requested an improved flexibility in terms of program initiation. Participants wanted the program to be adjusted to their respective gestational week. This request has not been accommodated, however. Mamma Mia starts in mid-pregnancy because it is considered optimal to promote relationship satisfaction and prenatal attachment early (but not too early when the risk of miscarriage is still high) when the goals are to prevent depression and enhance well-being. In consequence, the program initiation continues to be limited to gestational week 22.

### Limitations

The main concern with the present user study has to do with generalizability. Due to the use of a convenience sample the findings may not be representative for all mothers (eg, ethnically diverse users, fathers or partners, and users with lower socio-economic status). There was a substantial dropout from the treatment program in this study. However, treatment dropout is common to most Internet-based programs [67]. Particularly, for comprehensive multi-session interventions, like Mamma Mia (ie, for each session added), an opportunity for dropout is also added [33]. Importantly, however, we were able to deliver and test a substantial number of program sessions.

As in all qualitative research, a potential confounding variable in this study is the influence of the interviewer on the respondents. As the interviewees were aware that the interviewer had a professional interest in Mamma Mia, it may have influenced the responses in different ways. For instance, the participants may have wished to come across in a socially desirable manner, and hence were likely to give positive feedback. On the other hand, they may have felt that the interview was the perfect opportunity to communicate directly what could be improved to the developers of the program. As the interviewees all gave nuanced feedback, it is likely that they felt comfortable being honest in the interview setting. It is important to note that while additional nuances would probably be captured with a larger sample; the main themes described herein would remain [61]. Moreover, the themes from the interviews were in line with the findings from the quantitative data, which adds to the reliability of the findings.

It is of essence that participants are invited to use Mamma Mia when they are in gestational week 22, which is why follow-ups in hospitals and well-baby clinics are thought to be the best settings to recruit women. That way midwives and doctors can inform women of the program when they are close to gestational week 22. In the present study, the primary setting for recruitment was the hospital and well-baby clinics, and yet only 60% (62/103) of participants were recruited there. Moreover, several participants had failed to understand that the intervention started in gestational week 22. It is not clear what inhibited successful/efficient promotion of Mamma Mia in hospitals and well-baby clinics; nevertheless, it is clear that one has to find an efficient way to organize and deliver Mamma Mia which is unobtrusive to health care personnel. A more comprehensive monitoring of the recruitment process may have revealed the sticking points and thus been informative for further evaluation studies as well as real world implementation of Mamma Mia.

### Conclusions

We were able to deliver a substantial number of sessions, and the high scores on measures of usefulness, ease-of-use, credibility, unobtrusiveness, and user satisfaction suggest a high level of acceptance. Findings from the user survey and interviews were consistent, which add to the reliability of the results. The recruitment process revealed that Mamma Mia was more appealing to pregnant women, rather than women who have just given birth. This is advantageous and not surprising as the program is designed to follow women through both the pregnancy and postpartum months. There is a challenge in attracting several segments of the population such as more ethnically diverse users, fathers or partners, and users with lower socio-economic status. In order to further increase user acceptance of this and similar programs, making such programs more adaptable to individual needs and preferences seems to be a key factor. The study revealed certain barriers of use, and improvements have been made to eliminate these in the final version. Overall, the user acceptance of Mamma Mia was fairly good and our findings add to the feasibility of the program. We find it appropriate to proceed to the next step of evaluation: a randomized controlled trial to test the effect of Mamma Mia on well-being and postpartum depressive symptoms.

## Abbreviations

EPDS: Edinburgh Postnatal Depression Scale
PPD: postpartum depression
RBUP: Centre for Child and Adolescent Mental Health
